# Supplementary material for: Sleep Quality, Nutritional Habits, and Physical Activity in Pediatric Cancer Survivors: A Dyadic Analysis Approach
Source: Nutrients. 2025 Jan 11;17(2):250. doi: 10.3390/nu17020250 (PMC11767264; doi:10.3390/nu17020250)
Supplement: Supplementary file 1 [file nutrients-17-00250-s001.zip › nutrients-3399006-supplementary.pdf]

Supplementary Table S1: Post-Hoc Power Calculations for APIM Analyses

Post-Hoc Power Analysis

Model	# of Dyads	Actor-Partner Correlation	Error Correlation	Effect	Effect Size	Power
FAQ on PSQI	127	0.576	0.334	Actor Effect for Parent	0.179	69%
				Actor Effect for Child	0.072	17%
				Partner Effect for Parent	0.195	76%
				Partner Effect for Child	0.021	6%
				Difference in Actor Effects	0.107	17%
				Difference in Partner Effects	0.174	37%
				Average of Actor Effects	0.126	64%
				Average of Partner Effects	0.108	52%
PSQI on FAQ	127	0.368	0.534	Actor Effect for Parent	0.142	56%
				Actor Effect for Child	0.117	41%
				Partner Effect for Parent	0.050	12%
				Partner Effect for Child	0.229	93%
				Difference in Actor Effects	0.025	6%
				Difference in Partner Effects	-0.179	44%
				Average of Actor Effects	0.130	74%
				Average of Partner Effects	0.140	80%

Note. PSQI = Patient Sleep Quality Index; FAQ = Food and Activity Quality
